# Neglected dorsal dislocation of the scaphoid

**DOI:** 10.4103/0019-5413.50858

**Published:** 2009

**Authors:** Rajkumar S Amaravati, MJ Saji, HP Rajagopal, Gururaj

**Affiliations:** Department of Orthopedics, St. John's Medical College Hospital, Bangalore - 560 034, India

**Keywords:** Neglected scaphoid dislocation, proximal row carpectomy, scaphoid

## Abstract

Isolated dislocation of the scaphoid is very rare. A 45-year old male, industrial worker reported two and half months after injury with wrist pain and swelling on the dorsum of left wrist. He was diagnosed as neglected dorsal dislocation of scaphoid. Proximal row carpectomy with capsular interposition was done stabilizing the distal carpus on the radius using Kirschner wires. At-12 months follow-up the patient had good wrist function and was satisfied with the outcome of the treatment. We hereby report this neglected dorsal dislocation of scaphoid in view of rarity and discuss the various options for management.

## INTRODUCTION

Isolated scaphoid dislocation without fracture or dislocation of the associated carpal bones and fracture of radial or ulnar styloid is rare.^1^ Scaphoid dislocation can be due to isolated periscaphoid ligament injury or can be an element of complex carpal injury. The dislocation can be subdivided into subluxation, proximal dislocation or total dislocation.^2^ To the best of our knowledge only 13 cases of isolated dislocation of scaphoid have been reported in English literature. Out of these five were anterior, seven radial and one was a dorsal dislocation.^1^ Most dislocations were caused by violent dorsiflexion of the wrist while the hand was grasping a fixed object in ulnar deviation.^3^ The purpose of this case report is to describe this rare injury and the limitation of options available for treatment. A prior informed consent was obtained from the patient to publish the details concerning the case.

## CASE REPORT

A 45-year-old male industrial worker was admitted with pain and loss of motion of the left wrist of two and half months’ duration. His left wrist had been forced into dorsiflexion when he tried to prevent a 300-kg barrel from rolling over. He developed pain and swelling of the left wrist for which he received treatment from a local bone setter in the form of massage and splinting. He presented to us with unrelieved wrist pain and swelling on the dorsum of the left wrist.

**Figure 1:**
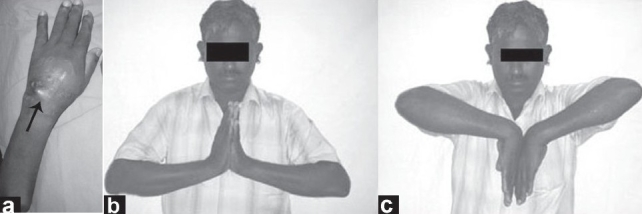
Clinical photograph (a) of (Left) wrist showing dorsally dislocated scaphoid [Arrow]. Clinical photograph (b and c) showing good wrist function at 12 months follow-up

**Figure 2:**
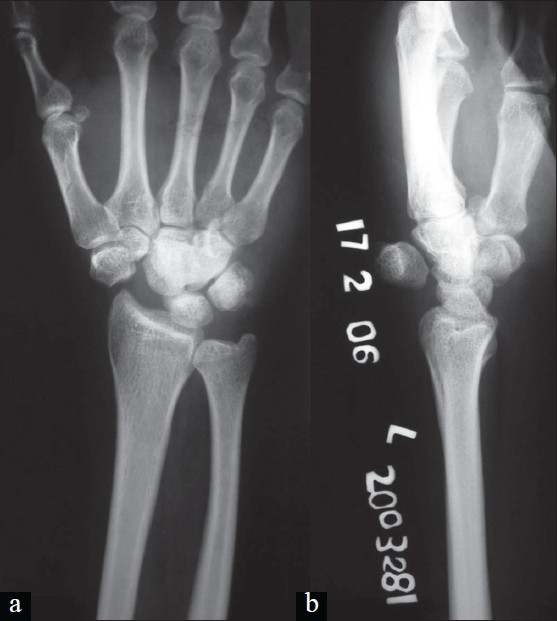
(a) Anteroposterior radiograph of left wrist shows missing scaphoid and loss of Gilula arc. (b) Lateral radiograph showing dorsally dislocated scaphoid.

**Figure 3:**
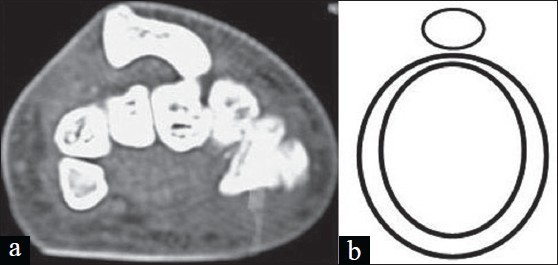
(a) CT scan showing dorsally dislocated scaphoid simulating “signet ring” (b) Line diagram representing signet ring.

On examination there was tender bony hard, swelling, measuring about 2.5 × 2cm, on the dorsal aspect of the left wrist [Figure 1a]. Anatomical snuff box was empty.^4^ Active and passive wrist movements were painfully restricted. Grip strength was weak. Clinically, a diagnosis of dislocated carpal bone was made. Anteroposterior (AP) and lateral radiographs of his left wrist revealed a mid-carpal radial luxation of the wrist with dorsal dislocation of the scaphoid, chip fracture of the trapezium and trapezoid [Figure 2]. Computerized tomography (CT) scans showed total dorsal dislocation of the scaphoid which appeared like a “signet ring” [Figure 3]. The magnetic resonance imaging (MRI) showed disruption of most ligaments around the scaphoid without avascular necrosis and intact triangular fibro-cartilage.

Under general anesthesia patient was positioned supine on the operating table and the affected arm was abducted on a hand table. Under tourniquet a mid-dorsal incision measuring 7-8 cm was made on the left hand. Dissection was carried down to the extensor retinaculum. Scaphoid was found dislocated dorsally through a rent in the extensor retinaculum devoid of any ligamentous attachment. It was excised after releasing it from the fibrous adhesions. Extensor pollicis longus and extensor pollicis brevis were identified and retracted. Dorsal capsule was further incised in line with the extensor tendon avoiding injury to the cartilage of the head of capitate. A distally based capsular flap was elevated. Articular cartilage of the head of capitate and lunate facet of the distal radius was found to be normal. The Steinmann pin was used as a joystick to remove lunate and triquetrum without injuring the volar capsule.

**Figure 4:**
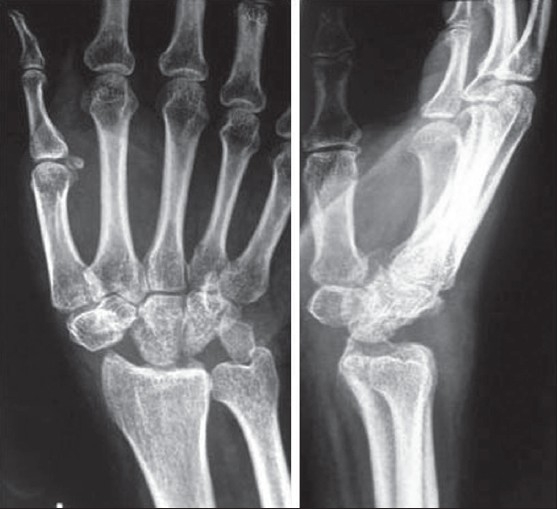
Anteroposterior and lateral radiograph of left wrist at 12 months follow-up shows alignment of carpus on distal radius

After excising the lunate and triquetrum, capitate was allowed to settle in the lunate facet of radius after interposing the capsular flap between the distal carpus and radius. A Kirschner wire was passed through hamate and trapezium into the distal radius under fluoroscopic guidance in functional position. The tourniquet was released and hemostasis was achieved. Wound was closed over a drain and compression bandage was applied. The kirschner wire was removed at the end of three weeks and the wrist was mobilized under supervision. At the latest follow-up of 12 months, the carpus was in satisfactory alignment on the distal radius [Figure 4]. The patient had good wrist score [Figure 1b, Figure 1c] on the clinical rating scale of Cooney *et al*. with 85 points and he was satisfied with the result.^5^

## DISCUSSION

Isolated dislocation of the carpal scaphoid was first reported in the English literature in 1930.^6^ An understanding of the anatomy and biomechanical properties of major periscaphoid ligaments is critical to the understanding of the dislocation of the scaphoid. Injury to the scaphotrapezial, scaphocapitate and scapholunate interroseous ligaments influences the chance of scapholunate instability after perilunate dislocation.^7^ The sequential pattern of failure of ligaments in dislocation of scaphoid could be radioscaphocapitate, scapholunate interroseous, long radiolunate and scaphotrapezial ligaments.^8^

The mechanism of injury attributed to scaphoid dislocation includes hyperextension and ulnar deviation of the wrist with intercarpal supination. Additional axial force from distal to proximal through the metacarpals may also cause disruption of the carpus.^6^ Isolated scaphoid dislocations can be classified as purely palmar or dorsal and radial palmar or radial dorsal.^9^ Leung *et al*.,^10^ described simple and complex injury patterns, primary versus secondary and partial versus total dislocation of scaphoid. Primary dislocation is due to direct injury, whereas secondary dislocation is persistent dislocation after closed reduction of the proximal carpus.^10^ Simple dislocation involves only the radioscaphoid and scapholunate articulations whereas complex dislocation also involves the capitohamate and middle ring metacarpal articulations. Partial dislocation has some soft tissue attachment (usually distally) and complete dislocation has loss of all soft tissue attachment.^10^

Initial diagnostic evaluation must include standard anteroposterior, lateral, oblique and radio-ulnar deviation radiographs. Arthrogram or MRI can be used to assess subtle ligament injury. Arthroscopy can be used as both diagnostic and therapeutic modality if imaging modalities are non-diagnostic.^2^ A CT scan, however, will help in assessing associated bony injury.

Treatment for acute scaphoid dislocation has ranged historically from closed reduction and casting to percutaneous fixation after closed reduction or open reduction and to open reduction and ligament reconstruction with internal fixation.^1^ In almost all cases, after proper treatment, the patients have returned to their previous activities with only moderate limitations. There is nearly universal loss of range of wrist motion.^1^ In cases where arthritic changes in a wrist are present due to carpal instability, best results were obtained with partial wrist arthrodesis with or without distal scaphoid excision.^11^ As one would expect the principal difficulty of small bone arthrodesis is nonunion.^11^ The present case presented to us late (two and a half months after injury) without any arthritic changes in the wrist. Hence proximal row carpectomy was done to provide functional range of motion and adequate grip strength. After proximal row carpectomy the range of flexion-extension and grip strength, expressed as percentage of the values on the contralateral side have averaged 62% and 80% respectively, with patient satisfaction ranging from 80% to 100%.^11^

Using cine radiography, it was found that motion of the capitate on the radius is translation, with a moving center of rotation. The combination of size mismatch between the capitate head and the lunate facet of the distal part of the radius and the translation motion of the wrist following proximal row carpectomy is responsible for the loss of the radio-capitate cartilage space. This, however, was found not to decrease the clinical function.^12^ The most common complication after scaphoid dislocation is residual rotatory subluxation. Median nerve compression and avascular necrosis have also been described.^13^,^14^ After proximal row carpectomy our patient was able to return to his work with slight loss of motion and was “satisfied” with the outcome.

Neglected total dorsal dislocation of the scaphoid is a rare entity. No firm conclusion can be reached from the result of a single case study, although it does suggest that proximal row carpectomy may provide benefits for a patient with neglected scaphoid dislocation. However, more cases with longer follow-up are needed to be studied.
